# Breast Ironing from the Perspective of Transcultural Nursing by Madeleine Leininger: A Narrative Review

**DOI:** 10.3390/nursrep14040269

**Published:** 2024-11-27

**Authors:** Rosa M. Cárdaba-García, Veronica Velasco-Gonzalez, Inés Cárdaba-García, Lucía Pérez-Pérez, Carlos Durantez-Fernández, Alba Muñoz-del Caz, Raúl Soto-Cámara, Marta Evelia Aparicio-García, Miguel Madrigal, Inmaculada Pérez

**Affiliations:** 1 Department of Nursing, Faculty of Nursing, University of Valladolid, Av. Ramón y Cajal, 7, 47005 Valladolid, Spain; rosamaria.cardaba@uva.es (R.M.C.-G.); lperezp@saludcastillayleon.es (L.P.-P.); carlos.durantez@uva.es (C.D.-F.); alba.munozdelcaz@uva.es (A.M.-d.C.); miguelangel.madrigal@uva.es (M.M.); maku@ioba.med.uva.es (I.P.); 2 Nursing Care Research (GICE), Faculty of Nursing, University of Valladolid, Av. Ramón y Cajal, 7, 47005 Valladolid, Spain; 3 Primary Care Management Segovia (SACYL), C. Santo Tomás, 9, 40002 Segovia, Spain; icardabag@saludcastillayleon.es; 4 Primary Care Management Valladolid West (SACYL), C/Dulzaina, 2, 47012 Valladolid, Spain; 5 Hospital Clínico Universitario de Valladolid (HCUV), Av. Ramón y Cajal, 3, 47003 Valladolid, Spain; 6 Department of Health Sciences, University of Burgos, C. Comendadores, s/n, 09001 Burgos, Spain; rscamara@ubu.es; 7 Emergency Medical Service of Castilla y León (SACYL), P.º del Hospital Militar, 24, 47007 Valladolid, Spain; 8 Instituto de Estudios Feministas, Universidad Complutense de Madrid, C. Isaac Peral, s/n, 28015 Madrid, Spain; meaparic@ucm.es; 9 IOBA (Institute of Applied Ophtalmobiology), University of Valladolid, Pº Belén, 17, 47011 Valladolid, Spain

**Keywords:** breast ironing, cultural practices, human rights, transcultural disease, violence against women

## Abstract

(1) Background: This article addresses the harmful traditional practice of breast ironing, which primarily affects girls and adolescents in several countries, particularly in Cameroon. The practice involves applying heat and pressure to developing breasts to delay their growth, with the goal of protecting girls from sexual abuse, early pregnancy, and forced marriages. While culturally accepted, breast ironing has severe physical, psychological, and social consequences, including damage to mammary glands, pain, infections, and potential long-term health complications. (2) Methods: A reflective analysis of the topic was conducted through a comprehensive search of various databases (PubMed, Scopus, Google Scholar, and CINAHL) following narrative review methodology. (3) Results: The practice is also examined through the lens of human rights and Madeleine Leininger’s transcultural nursing theory, which promotes respect for cultural traditions in healthcare. However, the authors question whether such a theory can justify practices that violate women’s physical integrity. (4) Conclusions: This study concludes that while cultural beliefs must be considered in healthcare, human rights and the elimination of violent practices, such as breast ironing, must take precedence. Educational campaigns and a more punitive approach in countries where this practice occurs are proposed as essential steps forward.

## 1. Introduction

Around the world, girls and women are subjected to harmful traditional practices [1]. According to the United Nations Children’s Fund (UNICEF), by 2023, approximately 640 million women worldwide will have been married as children, and at least 200 million will have undergone female genital mutilation [2]. These practices infringing on the dignity of girls and constitute a breach of human rights. On 10 December 1948, at the United Nations (UN) General Assembly in Paris, the Universal Declaration of Human Rights was adopted. Harmful practices against girls represent a form of violence deeply rooted in sex, gender, and age discrimination, which fundamentally contradicts the principles of the declaration [3]. Additionally, the sexual and reproductive rights of adolescent girls are infringed, contravening the fundamental principle of individual freedom to make decisions and freely exercise their sexuality [4].

The human rights of individuals, including sexual and reproductive rights, are framed within a global dimension implemented by international organizations but need to be adapted to align with local values and practices. The process of vernacularization, introduced by Peggy Levitt and Sally Engle Merry, translates international rights into locally meaningful frameworks using principles of social justice. According to these authors, social movements can frame societal problems as human rights issues, as was the case with gender violence. This concept also applies to other forms of violence against girls and women, including breast ironing [5].

In addition, the Sustainable Development Goals call for the elimination of all harmful practices that violate the rights of girls and women. UNICEF outlines actions necessary to achieve this goal, including raising awareness about harmful practices; developing and enforcing effective laws and policies to eradicate them; fostering cultural transformation; empowering women and girls to exercise their rights; providing prevention, protection, and quality care; engaging governments; and strengthening data collection systems [6].

Various forms of violence against women are widespread in Africa; however, assuming that these actions occur only on this continent reflects a Eurocentric and exclusively external perspective [7]. The focus should not be on where these harmful practices take place, but rather on why they occur and their consequences. It is important to note that such abuses are often culturally accepted and therefore frequently go unreported [6]. These practices include breast ironing, which significantly impacts the physical, psychological, and social well-being of women and girls [8]. However, breast ironing is not the only form of violence against girls and adolescents. Among the wide array of harmful practices prevalent worldwide are female genital mutilation, child and/or forced marriage, polygamy, bride kidnapping, honor killings, dowry or bride price violence, son preference, and female infanticide, among others.

Breast ironing involves the use of flat-surfaced materials that have been preheated over a fire and applied to the mammary glands. These materials, resembling irons, are kept in direct contact with the breast, pressing on it to slow its growth [9]. The most commonly used materials include flat stones for grinding or sharpening, sticks for crushing grain, mortar handles, burning charcoal, hot irons, or any other heated utensil that can be used to massage and compress the breast. The duration of the heat application and pressure is unclear, but it is typically done daily until breast growth stops. There are also known cases where breasts are bound, or a belt is placed around them to maintain continuous pressure [10].

These practices are carried out as part of cultural traditions intended to delay the onset of breast development (thelarche) in adolescent girls to protect them from male attention. The dilemma arises when this practice is viewed through the lens of transcultural nursing theory [11]. According to this theory, care should consider the health and illness beliefs of a society. If this is the case, breast ironing might appear justified, but a deeper analysis reveals more nuanced results [12].

Madeleine Leininger, the nurse who developed the theory of transcultural nursing in the 1960s, focused on “values, beliefs, and comparative cultural care practices of individuals or groups from similar or different cultures to provide universal and culture-specific nursing care practices in promoting health or well-being, or to help people cope with unfavorable human conditions, illness, or death in culturally meaningful ways” [13]. Her theory emphasizes respect for cultural traditions. For Leininger, nursing has a societal obligation to care for individuals, families, and communities. This care must integrate knowledge, technical skills, and cultural awareness, employing a holistic point of view [14]. Leininger laid the foundation for the development of transcultural nursing, as well as the theory of cultural care and culturally based healthcare. Transcultural nursing extends beyond mere awareness, utilizing nursing knowledge of cultural care to deliver culturally congruent and responsive care. This theoretical framework integrates nursing and anthropology, giving rise to what is known as ethno-nursing. Leininger explores the application of culturally congruent nursing care by deeply analyzing the meanings, beliefs, and practices of nursing from both emic and etic perspectives. Although it may initially seem complex, this theory has been recognized for its clarity. It emphasizes the concept of transculturality and qualitative nursing research to define what transcultural care should entail. It enables nurses to provide care grounded in a multicultural worldview, with the goal of delivering culturally specific nursing care [13,14,15].

Nursing in a multicultural and globalized world must adapt to society and to individuals within health and community contexts. This requires congruent nursing care that respects the values of an individual’s cultural background. It is important to recognize that cultures coexisting in the same environment influence one another, but this should never result in power imbalances. Women, in particular, are often affected by the intersectionality of various social categories, such as sex, gender, ethnicity, class, and sexual orientation, among others [16]. These factors influence women’s lives in numerous ways, from access to education and employment opportunities to experiences of economic and social inequality, including biases in healthcare. Cultural differences between a nurse and a migrant woman may present greater challenges than those between a nurse and a migrant man, as the latter is not similarly affected by gendered dynamics [7].

According to Leininger’s theory, nurses must be trained in the analysis of different cultures to better understand individuals’ experiences, particularly women subjected to violence, and thereby provide optimal care. Haraway’s concept of situated knowledge emphasizes the importance of embodied subjectivity, enabling nurses to empathize with women, avoiding judgment, and respecting them to provide the best care possible [17]. However, while judgment must be avoided, harmful practices cannot be condoned. Nurses have a responsibility to uphold human rights and comply with the laws of the country where they practice [18].

The aim of this study is to provide a general understanding of a harmful practice affecting adolescent girls, to identify the factors that drive it, to examine its implications for women’s health, and to analyze breast ironing from a human rights perspective and Leininger’s theory of transcultural care. This approach aims to increase awareness of breast ironing within the nursing profession, which constitutes the primary readership of this scientific journal. Furthermore, it seeks to highlight the importance of detecting this practice in order to intervene with a holistic and transcultural perspective in the care of women.

Certain harmful practices, such as female genital mutilation and forced marriage, have garnered significant attention in scientific literature. However, others, like breast ironing, have been less studied and are therefore largely invisible to society at large and to the scientific community in particular. This underscores the need for studies like this one to shed light on breast ironing and emphasizes the urgency of eradicating this practice.

## 2. Materials and Methods

To conduct a reflective analysis of the topic, a search for relevant documentation was carried out across various data sources. Primary sources included speeches by high-level United Nations officials, laws, academic papers, and scientific articles. Secondary sources used in this study were books and dictionaries. Finally, tertiary sources were consulted through a reverse search of the bibliographic references of scientific articles and the University of Valladolid’s library catalogue.

The documentation search was conducted in the databases Medline via PubMed, Scopus, Google Scholar, and CINAHL. These databases were chosen for their relevance to the health sciences field. Medline is likely the largest database for biomedical literature. Scopus offers access to emerging articles (preprints). Google Scholar allows for extensive searches when the literature on a topic is scarce, and CINAHL provides nursing articles, which form the core of this review.

**Figure 1:**
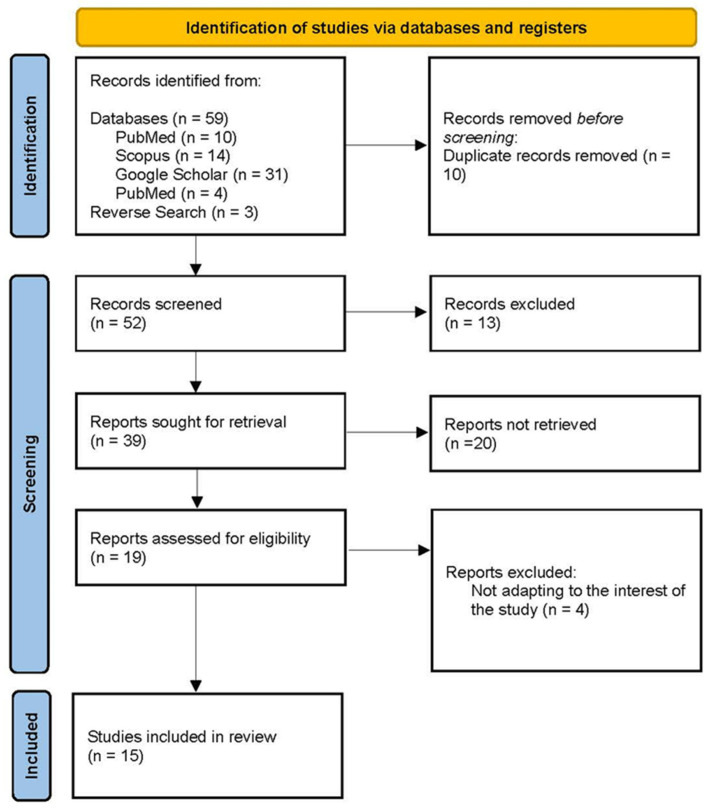
Flowchart diagram of studies included in review.

The database search was conducted using the following terms: breast ironing, cultural practices, transcultural care, human rights, and violence against women. These terms were combined using Boolean operators OR and AND. The search was limited to the period from 2014 to 2024, as selecting a shorter timeframe did not yield a sufficient number of scientific articles and books. A language filter was applied, including only texts in Spanish or English for which full access was available. The search took place on 3 August 2024. The initial search yielded 59 articles and books. An additional three articles were identified through a reverse search of the references cited in the scientific articles, bringing the total to 62. After removing 10 duplicate articles, 13 articles were excluded based on their abstracts. After reading the full text of the remaining articles and one book chapter, 15 studies (14 scientific articles and one book chapter) were selected for inclusion (Figure 1, Table 1).

## 3. Results/Discussion

### 3.1. Breast Ironing: A Cultural Practice and Its Impact on Women’s Health and Rights

Today, breast ironing is practiced in several African countries as a form of violence against adolescent girls, and it is considered one of the five most silenced forms of violence, according to UN data [30]. The country where this practice is most prevalent is Cameroon, where one in four women has undergone it, according to data from the German Agency for International Cooperation (GIZ). However, this practice is not confined to this region; it has also spread to Benin, Burkina Faso, the Central African Republic, Chad, Côte d’Ivoire, Guinea-Bissau, Guinea-Conakry, Kenya, Nigeria, Togo, South Africa, and Zimbabwe [1]. Additionally, cases of breast ironing among adolescent migrants from Africa have been reported in England, raising concerns among European health and social services [19,20,30].

In these cultures, thelarche (the onset of breast development) is viewed as problematic due to the potential consequences. Adolescent girls who begin to mature sexually are seen in their societies as desirable to men, putting them at risk of rape, early pregnancy, and HIV infection. To prevent these risks, breast ironing is performed on girls between the ages of 9 and 14. In most cases, the mother carries out the procedure, but it can also be done by another female relative. It is never performed by men [30,31]. The practice is usually kept secret between the girls and their mothers. Occasionally, traditional birth attendants or healers may perform the procedure, benefiting financially and gaining social status from it [1,21].

Breast ironing has a number of health effects on girls, including physical, psychological and social effects. As a result of this practice, women may suffer from consequences such as fibrosis, atrophy, damage to the lactiferous ducts, and subsequent breastfeeding difficulties, as well as pain, burns, and sexual dissatisfaction. It is not definitively known whether this practice is linked to the development of breast cancer [8,9,22]. Some authors suggest a potential link between breast ironing and breastfeeding difficulties. The psychological impact on women can be significant, as they may develop negative feelings toward their breasts or experience guilt if their breasts remain voluminous despite ironing. This practice can also lead to feelings of low self-esteem, as it constitutes an assault on the sexual autonomy of adolescent girls [23].

In certain regions, unsuccessful breastfeeding poses a serious risk to children, as breast milk is often the sole source of nutrition for newborns. This can place infants in life-threatening situations, increasing the risk of mortality [28].

From a feminist and social justice perspective, attention is drawn to the lack of male accountability, as men are often the perpetrators of sexual abuse against young girls. There is little emphasis on education around equality to prevent abuse. Instead, violence is perpetuated by women against adolescent girls to protect them from male abuse [32]. The goal of breast ironing is to control both a girl’s body and her sexuality. In patriarchal societies, female sexual behavior outside of marriage is perceived as dishonoring the family [23], and women are deprived of their physiological attributes and sexual autonomy [24].

The origins of breast ironing are not well-documented. According to Tapscott [31], it may have originated as a method to address breastfeeding issues, such as reducing pain and swelling during engorgement. The practice may have transitioned from rural to urban traditions, though the lack of documentation makes it difficult to fully understand its history [31,32]. Today, breast ironing is justified as a way to ensure girls continue their education rather than being forced into early marriage due to pregnancy [21].

The practice has significant physical, psychological, emotional, and social effects on women, both during and after the procedure [20,25].

### 3.2. Transcultural Nursing Theory: Promoting Integrated Care in a Diverse World

Engaging with the transcultural nursing paradigm involves understanding different cultures to provide meaningful and effective nursing care tailored to individuals’ cultural values and contexts. This requires respecting unique caregiving behaviors based on culture-specific beliefs and values. It also means moving away from ethnocentrism, which leads to the belief that one’s own cultural perspective is the only correct one, while others are viewed as incorrect [32,33].

Leininger emphasizes the diversity and universality of cultural care. While different societies display diversity in care practices, the universality of care as a central component of health systems is also evident [14].

This theory highlights the dual reality of nursing. On the one hand, it is part of the empirical health sciences, and on the other, it is rooted in social and human sciences, which consider the socio-cultural realities of patients. Transcultural nursing draws from both nursing and anthropology. This dualism is reflected in various aspects of nursing, such as the health–illness continuum, individual–community relationships, and techniques care [34,35].

Several factors have contributed to the development of this theory. The most important is the marked increase in global migration. Other factors include the rise of multicultural identities, the application of new technologies in care that may conflict with cultural values, the growing number of cultural conflicts worldwide, the increase in people traveling and working in different countries, the legal system’s recognition of the need to adapt care to cultural differences, the growing importance of women’s and children’s health in different societies, and the increasing acceptance of the environment’s influence on care [36].

### 3.3. Factors Perpetuating the Practice of Breast Ironing

Like many cultural practices, breast ironing is linked to religion, with Christian and animist communities being the most common practitioners. It is rarely practiced among Muslim groups [10]. Greater scientific knowledge and the devaluation of religious knowledge as non-scientific in the mid-19th century could have contributed to the elimination of such harmful practices, but this has not been the case [21].

The practice is deeply rooted in tradition and belief, and it is considered a ritual, an ancestral activity that takes place within families and is passed down through generations. It is justified to prevent greater harm. Tradition can legitimize violence against girls and violate human rights [33].

Adolescent girls are instilled with a fear of disappointing their families if they refuse to undergo breast ironing. Additionally, the practice is considered a taboo associated with sexuality, kept within the family and not discussed publicly [37].

The lack of reporting is a significant obstacle to stopping this practice. Reporting can lead to legal issues for women, especially mothers, who are often responsible for the procedure [1,38]. There is also a lack of education on human rights and ignorance of the consequences of breast ironing in communities where it is practiced. These factors contribute to the delay in eradicating the practice, along with the normalization of violence against women [26].

### 3.4. A Grave Violation of Women’s Human Rights and Physical Integrity

The Universal Declaration of Human Rights was proclaimed by the United Nations General Assembly in Paris on 10 December 1948 [39]. It was the first international document to outline the fundamental human rights that must be protected globally. Gender-based violence clearly constitutes a violation of these rights. Article 2 states that individuals cannot be deprived of rights or freedoms based on gender, and Article 5 asserts that no one shall be subjected to torture or cruel, inhuman, or degrading treatment or punishment. Following this declaration, the UN signed several international conventions, including the Convention on the Elimination of All Forms of Discrimination against Women (CEDAW) on 18 December 1979 [40]. While this convention takes a comprehensive approach to gender-based violence, it does not specifically address harmful traditional practices. Despite these and other recommendations, national legislation remains the most effective means of combating such practices.

In Cameroon, the country with the highest incidence of breast ironing, the 1996 Constitution recognizes human rights [41], and the 2016 Penal Code criminalizes female genital mutilation (FGM) [33]. However, breast ironing remains unpunished.

Educational and awareness-raising campaigns are needed to inform the public about the dangers of cultural practices like breast ironing [6]. Health education is essential to address this issue and should include comprehensive sex education targeting girls, boys, mothers, and fathers [27]. Despite current legislative measures, they appear insufficient, and stricter punitive approaches may be necessary [26,27,28,29,30].

### 3.5. Challenges of Transcultural Nursing in Addressing Breast Ironing

Like all nursing theories, Leininger’s transcultural nursing theory has strengths and weaknesses [42,43]. In this case, we will examine the limitations concerning breast ironing.

The ethno-nursing approach in this theory involves analyzing a foreign culture from an emic (insider’s) perspective, meaning the culture is evaluated based on how its members express or behave within it. The distinction between emic and etic (outsider’s) perspectives was introduced by the anthropologist Murdock, but it is not universally accepted, which presents a challenge. Additionally, this theory does not fully consider the dynamic nature of cultures, often portraying them as static without acknowledging the changes they undergo over time [44].

Although Leininger’s goal was to provide culturally coherent care, the theory is based on Eurocentric concepts of health and illness, which focus on efficiency in healthcare. Culture is not fully integrated into the conception of care, resulting in an unequal relationship between nurse and patient. Nurses understand the patient’s culture, but the patient may not understand the nurse’s, leading to potential mistrust [45].

At times, the model risks falling into determinism by assuming that all individuals from a particular culture will respond to illness in the same way. It tends to equate the group with the individual [42].

### 3.6. The Application of Transcultural Theory in the Nursing Care of Women Affected by Breast Ironing

The application of Leininger’s model to breast ironing involves providing nursing care rooted in an understanding of cultural preservation. This includes recognizing and respecting the protective intentions of mothers who perform this practice, while advocating for cultural accommodation through alternative methods that uphold cultural values but eliminate physical harm. Examples include awareness programs on girls’ rights and sexual health, and cultural restructuring by collaborating with community leaders to challenge the normalization of breast ironing. Educational and empowerment measures that align with cultural norms can further support these efforts [15,28].

A cross-cultural analysis of breast ironing requires acknowledging its cultural protective intent while emphasizing its contradictions with principles of physical and mental health. Initiatives led by local non-governmental organizations and international programs demonstrate that Leininger’s theory can be applied to address this practice with cultural sensitivity while mitigating unnecessary risks. This approach ensures the evolution of cultural practices into safer and more humane forms [15,46].

### 3.7. Community Nursing Intervention Proposal to Eradicate Breast Ironing

As indicated above, nursing works in the community. This is done through prevention, health promotion, and health restoration programs.

The objectives of a program to eliminate breast ironing would include the following:

1. Raising community awareness about the risks of breast ironing.
2. Empowering girls, adolescents, and women with knowledge about their rights and health.
3. Engaging men, community leaders, and religious figures to transform cultural norms.
4. Promoting cultural and social alternatives to protect girls from gender-based violence.

Key Components of the Proposal:

1. Community Sensitization and Education: Organize community talks in local centers to explain what breast ironing is, its harmful effects, and human and children’s rights. Include testimonies from survivors to share personal experiences. As concluding actions, use videos or social theater dramatizations to showcase alternatives to the practice and address its challenges.
2. Educational Programs in Schools: Integrate lessons on sexual and reproductive health, puberty, body acceptance, and the consequences of breast ironing into school curriculums. Conduct self-esteem and empowerment workshops, and provide teacher training on gender and health issues.
3. Empowerment of Mothers: Establish women’s support groups as safe spaces to discuss the motivations behind breast ironing and explore alternative protective measures for daughters. Offer training in income-generating skills to provide mothers with economic independence and reduce reliance on patriarchal norms.
4. Engaging Men and Community Leaders: Implement male leadership training programs to raise awareness among men. Facilitate intergenerational debates to encourage respectful discussions about traditions and their impact on girls’ health.
5. Creation of Support and Prevention Networks: Form community child protection committees to collaborate with health services and local authorities, creating a robust network for prevention and support.

This program proposal should be adapted to the context in which it is implemented. Some actions may be appropriate for one society, and some may not. Moreover, like all programs, it will need to be evaluated at both the process and outcome stages. Based on what has been achieved, it will be oriented in one way or another for the future [22].

Finally, the authors acknowledge several limitations in this study. The scarcity of high-quality literature on the subject prevented a systematic review of the literature. Nonetheless, a methodology similar to PRISMA guidelines was followed, making the findings reliable based on the studies analyzed.

## 4. Conclusions

Leininger’s theory undeniably contributes valuable insights into providing culturally sensitive care. However, in cases like breast ironing, there is a risk of normalizing harmful practices under the guise of cultural justification. Therefore, it is more appropriate to approach such issues from a human rights perspective rather than purely a cultural one. Culturally congruent care alone does not adequately address the need to prevent cultural practices that constitute gender-based violence. Nursing professionals have an ethical duty to avoid perpetuating harmful practices, regardless of their cultural origins. To support this responsibility, they are backed by a legislative framework, particularly in Europe, and can utilize the powerful tool of health education to advocate for the prevention of such practices.

## Figures and Tables

**Table 1:** Studies included in the review.

References	Study Aim	Study Design and Methods	Main Outcomes
[1] Amahazion F. (2021)	Provide a brief overview of the practice, discuss its underlying causes, analyze its health implications, and present recommendations	Narrative review	It requires legislative frameworks prohibiting the practice, comprehensive sexuality education that addresses gender-based violence, and social and cultural paradigm shifts in communities where it occurs.
[2] Sibanyoni E.K. (2022)	Book chapter	Narrative review	Education and awareness campaigns are recommended to inform and highlight the dangers of cultural practices that threaten the well-being of girls.
[8] Olusola Ajibade, B., et al. (2024)	Examine the cultural practice of breast ironing and its significant impact on the physical, psychological, and social well-being of women and girls	Systematic scoping review following PRISMA guidelines	Breast ironing is a harmful cultural practice with significant negative impacts on the health and well-being of women and girls. Comprehensive interventions, including legal measures, community education, and support services, are crucial to eliminating this practice and protecting the rights and health of those affected.
[9] Blanco, I.G., et al. (2015)	Not applicable	Letter to the editor	Perhaps progress is not being made in an organized manner from an educational and cultural standpoint regarding the erotic symbolism of a woman’s breasts. This may demonstrate men’s tight and abusive control over the female population, rooted in fear and insecurity when women are treated as equals in rights and responsibilities.
[19] Robinson F. (2019)	Determine the actions physicians should take if they suspect a case of breast ironing	Narrative review	The most effective approaches include community education and programs for young women, alongside health initiatives like those created by the Came Women and Girls Development Organisation.
[20] Simpson, H. (2018)	Investigate the practice of breast ironing in Africa and the UK	Narrative review	This public health issue has both physical and emotional implications, and the practice has spread to the UK. Raising awareness of breast ironing among health professionals is essential to support those affected and protect those at risk.
[21] Nyangweso M. (2022)	Explore rites of passage as they relate to health outcomes, such as breast ironing	Qualitative through interviews	These practices exemplify how rituals evoke health concerns in Africa. The rise of scientific knowledge and the classification of religious knowledge as non-scientific in the mid-nineteenth century led to the separation of physical healing from spiritual factors.
[22] Glover Williams, A., and Finlay, F. (2020)	Describe the characteristics of breast ironing and raise awareness of this this issue within the scientific community	Narrative review	When caring for girls from West Africa, health workers must be alert to the possibility that they may have been subjected to this practice.
[23] Gorar, M. (2022)	Analyze the practice of breast ironing	Narrative review	The purpose of breast ironing is, ultimately, to control both a girl’s body and sexuality. Since female sexual activities outside of marriage are perceived as a stain on a family’s name, this practice is rooted in patriarchal, honor-based values.
[24] Falana, T.C. (2022)	Analyze the practice of breast ironing from the perspective of universal human rights, particularly the right to personal integrity and sexual autonomy	Qualitative research, analytical expository method	This harmful practice is an abuse because it violates a girl’s right to full sexual autonomy and to possess the natural physiological attributes that adorn women. Strict laws and sanctions should be enacted to abolish and completely eradicate this barbaric and horrific mutilation.
[25] Nkwelle, N.N.N. (2019)	Examine the perceived long-term health outcomes of breast ironing and its effects on the quality of life for affected women	Quasi-experimental	Research revealed a strong link between women who experienced breast ironing and negative outcomes related to long-term physical, psychosocial, and emotional health, as well as a decline in quality of life during and after the practice.
[26] Chishugi, J., and Franke, T. (2016)	Determine the situation regarding sexual abuse of women in Cameroon	Qualitative: case study	Cameroon has adopted strategies aimed at eliminating violence against women, including ratifying international policies, revising penal codes, and supporting local and international initiatives that promote women’s rights. However, many of these laws remain largely symbolic.
[27] Djiemo, C. (2023)	Explore the experiences and perceptions of breast ironing in Cameroon from the perspectives of women, healthcare professionals, and community actors	Qualitative descriptive design	The practice of breast ironing is complex and contributes to both individual suffering and the deterioration of family relationships. Legislation is needed to ensure access to sex education for girls and boys, as well as for mothers and fathers.
[28] Fotabong, M., et al. (2023)	Investigate the prevalence, awareness, and adverse outcomes of breast ironing among Cameroonian women	Mixed method design involving a qualitative and cross-sectional study	Health education and the introduction of laws against breast ironing will go a long way in eliminating this harmful traditional practice.
[29] Ajayi Olofinbiyi, B., et al. (2024)	Examine the phenomenon of breast ironing within the broader context of gender-based violence in Africa, including its prevalence, socio-cultural roots, health consequences, social implications, prevention challenges, and intervention strategies	Narrative review	Political will and action are essential to preventing breast ironing in Africa. Leaders must prioritize legislation, allocate resources, and collaborate with stakeholders to eradicate this harmful practice and protect vulnerable individuals.

## Data Availability

Not applicable.

## Public Involvement Statement

No public involvement in any aspect of this research.

## Guidelines and Standards Statement

This manuscript was drafted against the enhancing transparency in reporting the synthesis of qualitative research (ENTREQ) for narrative reviews.
